# Evaluation and Comparison of the Inhibition Effect of Astragaloside IV and Aglycone Cycloastragenol on Various UDP-Glucuronosyltransferase (UGT) Isoforms

**DOI:** 10.3390/molecules21121616

**Published:** 2016-11-29

**Authors:** Ruixue Ran, Chunze Zhang, Rongshan Li, Bowei Chen, Weihua Zhang, Zhenying Zhao, Zhiwei Fu, Zuo Du, Xiaolang Du, Xiaolong Yang, Zhongze Fang

**Affiliations:** 1 Tianjin Key Laboratory on Technologies Enabling Development of Clinical Therapeutics and Diagnosis, School of Pharmacy, Tianjin Medical University, Tianjin 300070, China; ranruixue@tmu.edu.cn (R.R.); lirongshan@tmu.edu.cn (R.L.); chenbowei@tmu.edu.cn (B.C.); 2 Department of Colorectal Surgery, Tianjin Union Medical Center, Tianjin 300121, China; zhangchunzetj@163.com (C.Z.); woshifangzhongze@163.com (W.Z.); 3 Department of Pharmacy, Tianjin Union Medical Center, Tianjin 300121, China; clinpharmzhao@163.com; 4 Department of Toxicology, School of Public Health, Tianjin Medical University, Tianjin 300070, China; fffffzhiwei@163.com (Z.F.); zsjz2113@sina.com (Z.D.); 5 Key Laboratory of Cancer Prevention and Therapy, National Clinical Research Center for Cancer, Department of Pharmacy, Tianjin Medical University Cancer Institute and Hospital, Tianjin 300060, China; duxiaolang1988@126.com; 6 Department of Rehab and Sports Medicine, Tianjin Medical University, Tianjin 300070, China; yxl0925@sina.com

**Keywords:** astragaloside IV, cycloastragenol, UDP-glucuronosyltransferases (UGTs), herb–drug interactions

## Abstract

As one of the main active ingredients from Radix Astragali (RA), orally dosed astragaloside IV (AST) is easily transformed to sapogenin-cycloastragenol (CAG) by deglycosylation in the gastrointestinal tract. Because the potential adverse effects of AST and CAG remain unclear, the present study in this article was carried out to investigate the inhibition effects of AST and CAG on UDP-glucuronosyltransferases (UGTs) to explore potential clinical toxicity. An in vitro UGTs incubation mixture was employed to study the inhibition of AST and CAG towards UGT isoforms. Concentrations of 100 μM for each compound were used to initially screen the inhibitory efficiency. Deglycosylation of AST to CAG could strongly increase the inhibitory effects towards almost all of the tested UGT isoforms, with an IC_50_ of 0.84 μM and 11.28 μM for UGT1A8 and UGT2B7, respectively. Ulteriorly, the inhibition type and kinetics of CAG towards UGT1A8 and UGT2B7 were evaluated depending on the initial screening results. Data fitting using Dixon and Lineweaver–Burk plots demonstrated that CAG competitively inhibited UGT1A8 and noncompetitively inhibited UGT2B7. From the second plot drawn with the slopes from the Lineweaver–Burk plot versus the concentrations of CAG, the inhibition constant (*Ki*) was calculated to be 0.034 μM and 20.98 μM for the inhibition of UGT1A8 and UGT2B7, respectively. Based on the [I]/*Ki* standard ([I]/*Ki* < 0.1, low possibility; 1 > [I]/*Ki* > 0.1, medium possibility; [I]/*Ki* > 1, high possibility), it was successfully predicted here that an in vivo herb–drug interaction between AST/CAG and drugs mainly undergoing UGT1A8- or UGT2B7-catalyzed metabolism might occur when the plasma concentration of CAG is above 0.034 μM and 20.98 μM, respectively.

## 1. Introduction

*Radix Astragali* (RA), originating from the dried root of *Astragalus membranceus* (Fisch.) Bge. and *A. membranceus* (Fisch.) Bge. Var. *mongholicus* (Bge.) Hsiao [1], is popular in clinical practice for treating hypertension, heart disease, diabetic nephropathy, viral hepatitis, and other diseases [2]. Conventionally, RA is regarded as a sweet, warm-natured drug, without any distinct toxicity and side effects in regular dosages [3]. In traditional Chinese medicine (TCM), this herb is often combined with other herbs, such as *Angelica* and *Codonopsis pilosula*, in complex formulas such as Shenqifuzheng Injection and Bushenyiqi Decoction as adjuvant therapy in the treatment of cancers, strokes, and other diseases.

**Figure 1:**
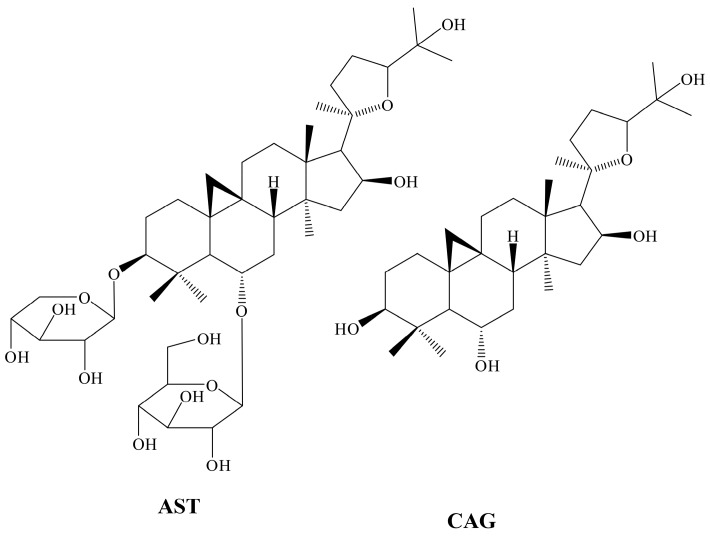
Chemical structure of astragaloside IV (AST) and cycloastragenol (CAG).

As one of the dominant bioactive components in RA, the astragaloside IV (AST) (Figure 1) level is required to determine the Quality Control (QC) of RA slices and extracts [4]. Moreover, AST itself is currently under development as a potential New Molecular Entity because of its cardiac, antihypertension, antiviral, antioxidant, and immunity effects [5]. Studies focusing on the pharmacokinetics and bioavailability of astragaloside IV suggest that astragaloside IV has low bioavailability after oral administration, which is only 2.2%–3.7% [6,7] in rats and 7.4% in dogs [8]. Apart from physicochemical characteristics such as high molecular weight, high hydrogen-bonding capacity, high molecular flexibility, poor membrane permeability, poor absorption through the gut, and limited metabolism by intestinal microflora may contribute to the poor bioavailability of AST [9]. However, AST undergoes intestinal bacterial biotransformation, and the main resultant metabolite, cycloastragenol (CAG) (Figure 1), can be more readily absorbed to reach systemic circulation. When AST was orally administered to rats, CAG presented as the main component in plasma following AST, and the area-under-the-curve (AUC_0-∞_) were 88.60 ± 9.66 (CAG) and 452.28 ± 43.33 nM·h (AST) [1]. 

Although much research has been carried on the in vitro and in vivo metabolism profiles of AST and CAG, only a few articles that deal with metabolism enzymes elucidate the potential toxicity and herb-drug interactions. Shan et al. [10] reported that AST was a competitive inhibitor of CYP2C9 and a non-competitive inhibitor of CYP3A4 in an in vitro study. Another study performed by Zhang et al. [11] found that AST exerted obvious inhibitory effects on CYP1A2 activity in rats, using theophylline as the probe. However, compared with phase I metabolizing enzymes, no research has been conducted on the interaction of AST and CAG with phase II conjugating enzymes. 

Human UDP-glucuronosyltransferase (UGT) enzymes are one of the most important phase II metabolizing enzymes, predominantly catalyzing the glucuronidation reactions of endobiotics, drugs and xenobiotics, generally making these molecules more amended to biliary and renal excretion. The disposition and clearance of drugs may be significantly changed when UGT are modulated by co-administrated drugs or herbal medicines. For example, fluconazole itself is not metabolized by UGT but alter pharmacokinetic parameters of co-administrated zidovudine in AIDS patients, because the glucuronidation of zidovudine by UGT2B7 is strongly inhibited by fluconazole [12]; indinavir [13] and sorafenib [14] can disturb the activity of UGT1A1, resulting in the inhibition of bilirubin gulucuronidation; atractylenolide I and III have been proven to specifically inhibit UGT2B7, thus affecting drugs undergoing UGT2B7- catalyzed metabolism [15]. 

Since the drug-drug interaction is considered as an important cause of adverse drug reactions, the present study aims to determine the inhibition behavior of AST and CAG towards UGT, and indicate the potential herb-drug interaction induced by UGT inhibition. Thus, the present study evaluated and compared the inhibitory effects of AST and CAG towards various UGT isoforms simultaneously. The experimental results should help clinic doctors and pharmacologists to re-evaluate the risk-benefits ratio when RA or AST are co-administered with drugs mainly metabolized by the same UGTs.

## 2. Results

### 2.1. Comparison of Inhibition Effect of AST and CAG

**Figure 2:**
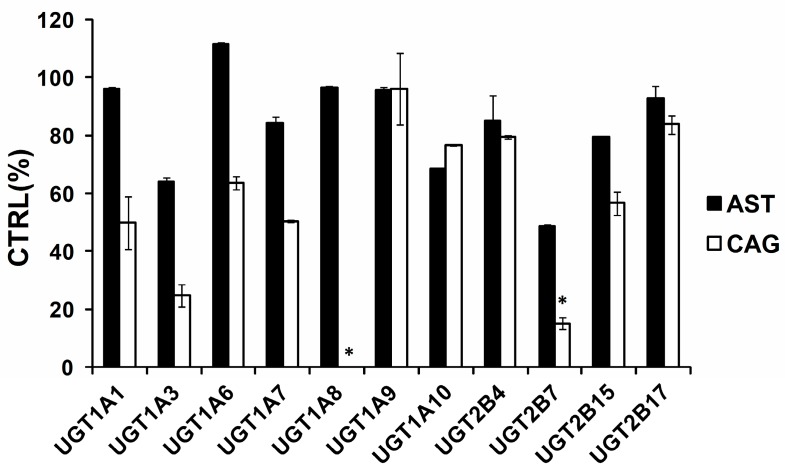
Screening the inhibition of UGT isoforms by 100 μM AST and CAG. 4-methylumbelliferone (4-MU) was used as a probe substrate for recombinant human UGT1A1, UGT1A3, UGT1A6, UGT1A7, UGT1A8, UGT1A9, UGT1A10, UGT2B4, UGT2B7, UGT2B15, and UGT2B17, and data are shown using mean value plus SD. * *p* < 0.05.

For the inhibition screening of compounds on the activity of drug-metabolizing enzymes (DMEs), 100 μM has been widely accepted to be the most optimal initial screening concentrations [16,17,18]. Based on this previous literature, we also selected this concentration as the initial screening concentration. As exhibited in Figure 2, 100 μM AST inhibited the activity of UGT1A1, 1A3, 1A6, 1A7, 1A8, 1A9, 1A10, 2B4, 2B7, 2B15, and 2B17 by 3.8%, 35.9%, −11.4%, 15.8%, 3.7%, 4.2%, 31.4%, 14.9%, 51.5% , 20.5%, and 7.2%, respectively. 100 μM CAG inhibited the activity of UGT1A1, 1A3, 1A6, 1A7, 1A8, 1A9, 1A10, 2B4, 2B7, 2B15, and 2B17 by 50.2%, 75.2%, 36.3%, 49.7%, 100.0%, 4.0%, 23.3%, 20.7%, 84.9%, 43.4%, and 16.3%, respectively. These results show that the deglycosylation of AST to CAG strongly increased the inhibitory effects towards almost all of the tested UGT isoforms.

### 2.2. Inhibition Type and Kinetics of CAG Towards UGT1A8 and UGT2B7

**Figure 3:**
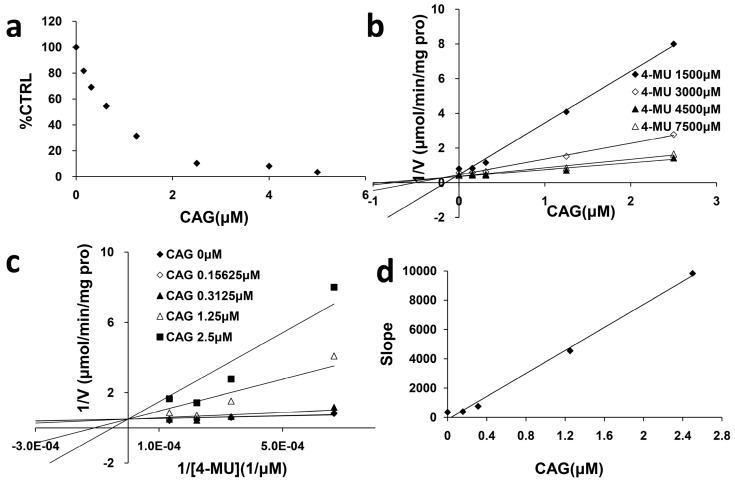
Determination of inhibition type and parameters (*Ki*) of CAG towards UGT1A8. (**a**) Dose-dependent inhibition of CAG towards UGT1A8; (**b**) Dixon plot of inhibition of CAG towards UGT1A8; (**c**) Lineweaver-Burk plot of inhibition of CAG towards UGT1A8; (**d**) Second plot of inhibition of CAG towards UGT1A8. The data point represents the mean value of duplicate experiments.

**Figure 4:**
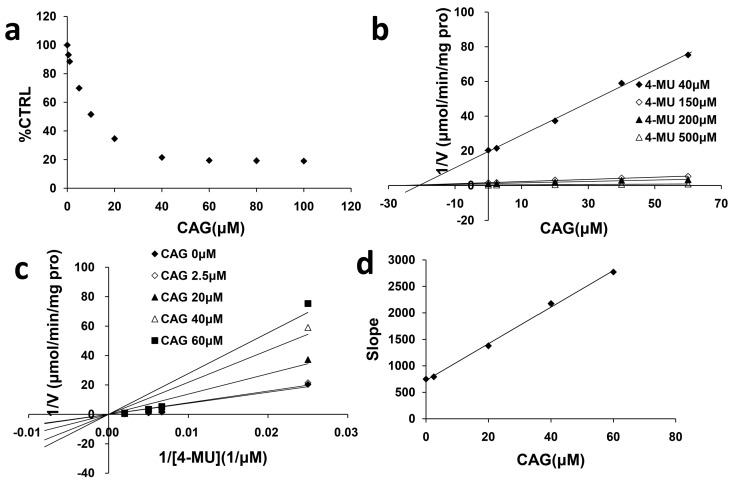
Determination of inhibition type and parameters (*Ki*) of CAG towards UGT2B7. (**a**) Dose-dependent inhibition of CAG towards UGT2B7; (**b**) Dixon plot of inhibition of CAG towards UGT2B7; (**c**) Lineweaver-Burk plot of inhibition of CAG towards UGT2B7; (**d**) Second plot of inhibition of CAG towards UGT2B7. The data point represents the mean value of duplicate experiments.

As exhibited in Figure 2, 100 μM CAG strongly inhibited the activity of UGT1A8 and UGT2B7 by 100% and 84.9%, respectively. As shown in Figure 3a and Figure 4a, CAG inhibited UGT1A8 and UGT2B7 in a concentration-dependent manner with an IC_50_ of 0.84 μM and 11.28 μM, respectively. To further explore the inhibition information, we evaluated the inhibition type and parameters of CAG towards UGT1A8 and UGT2B7. CAG competitively inhibited UGT1A8 and non-competitively inhibited UGT2B7, as demonstrated via Dixon plot (Figure 3b and Figure 4b) and Lineweaver–Burk plot (Figure 3c and Figure 4c). The second plot (Figure 3d and Figure 4d) was drawn with the slopes from the Lineweaver-Burk plot versus the concentrations of CAG, and the inhibition constant (*Ki*) were calculated to be 0.034 μM and 20.98 μM for the inhibition of CAG towards UGT1A8 and UGT2B7, respectively. 

## 3. Discussion

Inhibitory drug-drug interactions (DDIs) are considered as an important origin of adverse effects and have led to the withdrawal of several approved drugs from the market, which means it is not only a clinical problem, but also a potential economic loss for the pharmaceutical industry. Thus, it is quite necessary to profile and evaluate potential inhibitory effects of drug candidates through in vitro experiments, which is important for prediction of drug-drug interactions.

In our research, we studied the inhibitory effects of AST and CAG on 11 UGT isoforms and came to the conclusion that deglycosylation of AST to CAG can strongly increase the inhibitory effects towards almost all tested UGT isoforms. Results from research carried out by Guo et al. [19] showed that the aglycone liquiritigenin and glycyrrhetic acid exhibited stronger inhibition than their saponin, liquiritin, and glycyrrhizin. Cao et al. [20] evaluated the inhibitory effect of UGT1A7 by glucoaurantio-obtusin and aurantio-obtusin, indicating the importance of deglycosylation process for strengthening the inhibitory effect of glucoaurantio-obtusin towards UGT1A7. Besides, the deglycosylation of saponin to aglycone can also strongly increase the inhibitory effects towards CYPs. Liu et al. [21] found that naturally occurring ginsenosides exhibited no inhibition or weak inhibition against human CYPs, while their main intestinal metabolites demonstrated a wide range of inhibition of the P450-mediated metabolism. However, different from the above articles, it is interesting that the intestinal metabolites of icariin exhibited a different inhibition profile compared with icarrin [22]. Structural insights into UGTs and glycosylation mechanism may give us a proper explanation to this difference. In the structures of UGTs, the acceptor binding pocket is adjacent to the donor binding site, mainly consisting of residues in the *N*-terminal domain and some residues in the C-terminal domain, formed by several helices and loops. These regions are highly varied in these UGTs, thus making the interaction between UGTs and donor-glycosides and the interaction between UGTs and receptor-products of deglycosylated glycosides different [23].

As exhibited in Figure 2, 100 μM CAG strongly inhibited the activity of UGT1A8 and UGT2B7 by 100% and 84.9%. Although liver is considered to be the main organ of drug metabolism for derivative generation, the intestinal mucosa, as the first exposure site to orally dosed chemical substances, may play an important role in the first-pass metabolism of xenobiotics [24]. UGT1A8 is expressed in the mucosa of both the small intestine and the colon; therefore, UGT1A8 plays important roles in the first step of inactivation and detoxification in vivo. UGT1A8 shows higher catalytic activities toward the glucuronidation of catechol estrogens, coumarins, flavonoids, anthraquinones, and phenolic compounds [25]. Besides UGT1A8, CAG had strong inhibition towards UGT2B7, which is arguably the most important isoform because it contributes to the glucuronidation of 35% of clinically used drugs and endogenous compounds including bile acid, fatty acids, and steroids [26]. When considering clinically relevant DDIs involving UGTs, the enzyme that is most commonly implicated is UGT2B7 [27]. 

In practice, the [I]/*Ki* ratio is used to predict the likelihood inhibitory drug-drug interactions. When assessing in vivo interaction potential, [I] represents the mean steady-state Cmax value following the administration of the highest proposed clinical dose [28]. When CAG was co-administrated with drugs mainly undergoing UGT1A8- or UGT2B7-catalyzed metabolism, in vivo herb-drug interaction could have occurred when the mean steady-state Cmax of CAG was above 0.034 μM and 20.98 μM, respectively.

Although speculation on the clinical relevance of in vitro observations of inhibition of glucuronidation are relatively common, appropriate mechanistic studies attempting to link in vitro inhibition with influence on AUC in humans are rare. In fact, high risk occurs only when the AUCi/AUC ratio is above 5; however, observed AUCi/AUC ratios for glucuronidated drugs co-administered with UGT inhibitors are typically less than 2, thus indicating a low possibility of inhibitory drug-drug interactions caused by UGTs [29]. In view of the low levels of AST and its intestinal metabolite-CAG in blood, AST/CAG is impossible to cause a clinically significant DDI through the inhibition of hepatic glucuronidation after oral administration. However, in our research, CAG extremely strongly inhibited the activity of UGT1A8, an extrahepatic isoform. UGT1A8 may make great contributions to the first-pass metabolism of orally administered drugs that undergo glucuronidation. Therefore, AST/CAG may exert an influence on the glucuronidation and first-pass metabolism of some drugs orally administered, thus predicting inhibitory DDI potential.

In conclusion, the present study investigated the inhibitory potential of AST and CAG towards 11 UGT isoforms, to predict the possible herb-drug interactions between RA/AST and co-administrated drugs. The IC_50_ of CAG towards UGT1A8 and UGT2B7 was calculated to be 0.84 μM and 11.28 μM, respectively. The inhibition constants (*Ki*) were calculated to be 0.034 μM and 20.98 μM for UGT1A8 and UGT2B7, respectively. The experimental data will be of considerable referential importance when RA or AST/CAG is co-administered with drugs mainly metabolized by UGT1A8 or UGT2B7 for consequent excretion.

## 4. Materials and Methods

### 4.1. Materials and Reagents 

AST and CAG were purchased from Tianjin Yousi Pharma Ltd. (Tianjin, China), with the purity both above 98%. 4-Methylumbelliferone (4-MU), 4-MU-b-d-glucuronide (4-MUG), 7-hydroxycoumarin, Tris-HCl, and uridine-50-diphosphoglucuronicacid (UDPGA) (trisodium salt) were purchased from Sigma-Aldrich (St Louis, MO, USA). Recombinant human UGT supersomes (UGT1A1, 1A3, 1A6, 1A7, 1A8, 1A9, 1A10, 2B4, 2B7, 2B15, and 2B17) expressed in baculovirus-infected insect cells were obtained from BD Gentest Corp. (Woburn, MA, USA). All other reagents were of high-performance liquid chromatography (HPLC) grade.

### 4.2. Inhibition of UGT Activity Assay

The in vitro incubation experiment was carried out according to previously literature [15]. In brief, a typical 200 μL incubation mixture contained various recombinant UGT isoforms, 5 mM UDPGA, 5 mM MgCl_2_, 50 mM Tris-HCl buffer (pH = 7.4), and various concentrations of 4-MU. 4-MU was used as a non-selective substrate of UGTs. There was a 5 min pre-incubation step at 37 °C before the reaction was initiated by the addition of UDPGA. 4-MU and inhibitors were previously dissolved in DMSO, and the total concentration of DMSO was 1% (*v*/*v*). The reactions were continued at 37 °C for 120 min for UGT1A1, UGT1A10, UGT2B4, UGT2B7, UGT2B15, and UGT2B17, 75 min for UGT1A3, and 30 min for UGT1A6, UGT1A7, UGT1A8, and UGT1A9, respectively. Reactions were terminated by the addition of 100 μL of acetonitrile with 7-hydroxycoumarin (100 μM) as the internal standard. The incubation mixtures were then centrifuged at 12,000× *g* for 10 min. 

### 4.3. Analytical Methods

An amount of 2 μL of supernatant was injected into the UPLC system for analysis. A Waters ACQUITY UPLC System equipped with an UV detector was used to analyze the samples, and the separation of all compounds was carried out using an ACQUITY UPLC^®^ BEH C18 (2.1 mm × 100 mm, 1.7 μm, Waters, Milford, MA, USA) at a flow rate of 0.2 mL/min and an UV detector at 316 nm. The mobile phase consisted of ultrapure water containing 0.5% formic acid (*v*/*v*) (A) and acetonitrile (B). The following gradient condition was used: 0–3.5 min, 10%–65% B; 3.5–4.0 min, 65% B; 4.0–9.0 min, 10% B. The retention times for 4MUG, 7-hydroxycoumarin (internal standard) and 4MU were 2.6, 3.2, and 3.5 min, respectively.

### 4.4. Data Analysis

To calculate the standard curve, 0.1–100 μM 4-MUG was used by drawing the peak area ratio of 4-MUG/internal standard towards the concentration range of 4-MUG, with a linear correlation coefficient r^2^ over 99% (1/r^2^). We force the calibration equation through zero because we assume no HPLC response when no metabolite exists. The fitting equation was y = 0.0282x. The detection and quantification limits were obtained via signal-to-noise ratios of 3:1 and 10:1, respectively. The LOD and LOQ were 0.015 μM and 0.03 μM, respectively. The accuracy and precision of the back-calculated values for each concentration were less than 5%. All experiments were separately performed in duplicate three times. The determination of IC_50_ was calculated using Probit analysis in SPSS11.5 (SPSS, Chicago, IL, USA). The Student’s *t*-test was adopted at a significance level of *p* < 0.05 to determine statistically significant differences among experimental groups.

### 4.5. Inhibition Kinetics

The inhibition type and Kinetics were determined for the inhibition of CAG towards UGT1A8 and UGT2B7. The half inhibition concentration (IC_50_) values were determined using various concentrations of CAG (0.15625, 0.3125, 0.625, 1.25, 2.5, 4, and 5 μM for UGT1A8 and 0.5, 1, 5, 10, 20, 40, 60, 80, and 100 μM for UGT2B7). Inhibition constant (*Ki*) were determined utilizing various concentrations of 4-MU (1500, 3000, 4500, and 7500 μM for UGT1A8 and 40, 150, 200, and 500 μM for UGT2B7) in the absence (control) and presence of different concentrations of CAG (0.15625, 0.3125, 1.25, and 2.5 μM for UGT1A8 and 2.5, 20, 40, and 60 μM for UGT2B7). Dixon and Lineweaver–Burk plots were employed to determine the inhibition type, and the second plot of the slopes from the Lineweaver–Burk plot versus the compound concentrations was utilized to calculate the *Ki* value.

### 4.6. In Vitro–In Vivo Extrapolation (IVIVE)

In vitro–in vivo extrapolation (IVIVE) was performed using the following equation:

AUCi/AUC = 1 + [I]_in vivo_/*Ki*

The terms are defined as follows: AUCi/AUC is the predicted ratio of the in vivo exposure of the xenobiotics or endogenous substances with or without the co-exposure of CAG. [I]_in vivo_ is the in vivo exposure concentration of CAG, and the *Ki* value is the in vitro inhibition constant.
